# Comparative Study of the Impact Wedge-Peel Performance of Epoxy Structural Adhesives Modified with Functionalized Silica Nanoparticles

**DOI:** 10.3390/polym13030469

**Published:** 2021-02-02

**Authors:** Gyeong-Seok Chae, Hee-Woong Park, Kiok Kwon, Seunghan Shin

**Affiliations:** 1 Green Chemistry & Materials Group, Korea Institute of Industrial Technology (KITECH), Cheonan, Chungnam 31056, Korea; sugug12@kitech.re.kr (G.-S.C.); binufamily@kitech.re.kr (H.-W.P.); kioks@kitech.re.kr (K.K.); 2 Department of Green Process and System Engineering, University of Science & Technology (UST), Daejeon, Chungnam 34113, Korea

**Keywords:** epoxy toughening, silica nanoparticle, surface modification, impact wedge-peel test, structural adhesive

## Abstract

Epoxy structural adhesives have strong adhesion, minimal shrinkage and high thermal and chemical resistance. However, despite these excellent properties, their high-energy impact resistance should be improved to satisfy the increasing demands of the automotive industry. For this reason, we used four types of silica nanoparticles with different surface groups, such as polydimethylsiloxane (PDMS), hydroxyl, epoxy and amine groups, as toughening agents and examined their effect on the glass transition temperature (*T_g_*), crosslinking density and phase separation of epoxy structural adhesives. High-energy impact resistance, mode I fracture toughness and lap shear strength were also measured to explain the effect of surface functional groups. Silica nanoparticles with reactive functional groups increased the mode I fracture toughness of epoxy structural adhesives without sacrificing the crosslinking density. Although the mode I fracture toughness of epoxy structural adhesives could not clearly show the effect of surface functional groups, the dynamic resistance to cleavage obtained by impact wedge-peel tests showed quite different values. At a 0.3 vol% content, epoxy-functionalized silica nanoparticles induced the highest value (40.2 N/mm) compared to PDMS (34.1 N/m), hydroxyl (34.9 N/mm), and amine (36.1 N/m). All of these values were significantly higher than those of pristine epoxy structural adhesive (27.7 N/mm).

## 1. Introduction

Structural adhesives are high-strength permanent adhesives for structural materials such as metals and plastics, and they also have high impact strength, as well as high peel, bending, and fatigue strength [1]. In recent years, there has been an increasing demand for structural adhesives in the automotive, aviation and ship industries, where vehicle weight reduction has become important to reduce energy consumption and carbon emissions. As structural adhesives, epoxy-based adhesives are commonly used as structural adhesives due to their strong adhesion, minimal shrinkage, and high thermal and chemical resistance [2,3]. However, their impact properties should be improved to expand the applications [4].

Various studies have been conducted to overcome the brittleness of epoxy resins by adding toughening agents. First, the following were tried: butyronitrile-based liquid rubbers, such as amine-terminated butyronitrile (ATBN), carboxylic acid-terminated butyronitrile (CTBN), and epoxy-terminated butyronitrile (ETBN) [5,6,7,8,9,10,11,12,13,14]. Thermoplastic elastomers, such as olefin, urethane, and amide, are commonly used as toughening agents [15,16,17,18]. In both cases, the tlfexcess number of toughening agents decreases the glass transition temperature (*T_g_*) and other mechanical properties. Second, inorganic fillers, such as glass beads, alumina, and silica, are used as hard toughening agents [19,20,21]. These inorganic fillers can lessen *T_g_* reduction, although their toughening effects are inferior to those of liquid rubbers and thermoplastic elastomers.

Among inorganic fillers, silica nanoparticles have been actively studied, and many previous works have reported that they also strengthen the polymer matrix to reduce the shrinkage of the curing and thermal expansion coefficient and improve the adhesive properties, abrasion resistance, and corrosion resistance of the polymer matrix [22]. In addition, studies on improving the impact resistance of epoxy by surface modification of silica nanoparticles have been conducted [23,24,25]. Ragosta et al. studied the chemical interactions between epoxy and silica nanoparticles and analysed their stiffness and toughness improvement [23]. They found that the epoxide groups and silanol groups reacted, which improved the adhesion properties of the polymer matrix. Wichmann et al. reported an improvement in fracture toughness by substituting the hydroxyl group of fumed silica with an epoxide or an amine group [24]. According to their report, 0.5 vol% epoxy-modified silica improved the fracture toughness by 54% compared to an epoxy without silica. Dittanet and Pearson studied the effect of silica particle size (23, 74 and 170 nm) and its content on epoxy toughening [25]. They reported that the toughness of epoxy is affected by silica content but not particle size.

A common measurement method for the impact strength of epoxy resins is the Izod or Charpy impact test [26,27,28,29,30]. However, these are not suitable methods for evaluating automotive structural adhesives, which should withstand huge impacts. Therefore, in 1993, the ISO 11343 standard, including an impact wedge-peel test (IWP test), was adopted by the automotive industry to evaluate the impact properties of structural adhesives [31]. However, there have been few papers on the impact resistance of epoxy structural adhesives measured by IWP tests. Blackman et al. reported the impact resistance of epoxy structural adhesives evaluated by the IWP test for the first time [32]. They assessed the impact resistance of epoxy structural adhesives by adjusting the measurement temperature and impact rate and compared their fracture energy with those obtained by the T-peel test. Thereafter, Taylor et al. evaluated the impact resistance of commercialized epoxy structural adhesives with the IWP test [33]. Recently, Back et al. evaluated the impact resistance of epoxy foam adhesive with respect to CSR content [34]. They also synthesized a polyol containing phosphorus and examined the impact resistance and flame retardancy of epoxy structural adhesives by varying the polyol content [35].

In our previous study, we modified epoxy structural adhesives with soft elastomers, such as phenol-terminated polyurethane polyol (PTPU) and CTBN, and compared their impact resistance using the IWP test [36]. However, a previous study showed that PTPU interferes with the crosslinking reaction and decreases *T_g_* and crosslink density. Therefore, in this study, silica nanoparticles were used to improve high-energy impact resistance without sacrificing thermomechanical properties. As previously mentioned, the effects of surface functional groups of silica nanoparticles on the fracture toughness of epoxy resin have already been reported [23,24]. However, there are few studies on the high-energy impact properties of epoxy structural adhesives using the IWP test, and to the best of our knowledge, there is no report on the effect of silica surface functional groups on the fracture toughness and high-energy impact properties of epoxy structural adhesives. We prepared four types of silica nanoparticles with different surface groups: polydimethylsiloxane (PDMS), hydroxyl, epoxy and amine groups, and then fabricated epoxy structural adhesives with varying volume contents. Single-edged notch bending (SENB) and IWP tests were performed to observe the effect of the silica surface groups on the fracture toughness and high-energy impact properties. To analyse the results of the IWP and lap shear test, the *T_g_s* and crosslinking density of the epoxy structural adhesives were measured. Their fracture surface morphologies and silica distribution were also examined here.

## 2. Experiments

### 2.1. Materials

Epoxy resin (YD-128, EEW = 184–190 g/eq, viscosity = 11.5–13.5 Pa·s, specific gravity = 1.17) based on diglycidyl epoxy of bisphenol A (DGEBA) was supplied by Kukdo Chemical Company (Seoul, South Korea), and PTPU (DY-965) was purchased from Huntsman Corporation (The Woodlands, TX, USA). Dicyandiamide (DICY) hardener (Dyhard 100, powder, particle size ≤40 µm) was purchased from AlzChem Group (Trostberg, Germany), and *N*,*N*-dimethyl-*N*-phenylurea accelerator (Omicure U-405) was purchased from DyStar Group (Singapore, Singapore).

Hydrophilic fumed silica (Aerosil 200, renamed FS-OH) was purchased from Evonik (Essen, Germany). 3-Glycidyloxypropyl trimethoxysilane (GPS, ≥98%), 3-aminopropyl trimethoxysilane (APS, ≥97%), toluene (anhydrous, 99.8%) and ethanol were supplied by Sigma Aldrich (St. Louis, MO, USA) and used for the surface modification of fumed silica without further purification. Hydrophobic fumed silica (Cabosil TS-720, renamed FS-PDMS), which has a polydimethylsiloxane (PDMS)-treated surface, was purchased from Cabot (Boston, MA, USA).

Hydrobromic acid (48 wt% in H_2_O), acetic acid (glacial, ≥99%), potassium hydrogen phthalate (KHP) and 4-methyl-2-pentanone (MIBK) were purchased from Sigma Aldrich (St. Louis, MO, USA) and used for the titration of modified silica nanoparticles.

### 2.2. Surface Modification of Silica Nanoparticles

The surface modification of silica nanoparticles was performed using two types of organosilanes (GPS, APS) to introduce epoxy and amine groups on the silica surface. For the surface modification, 10 g of fumed silica nanoparticles were activated for 24 h in a vacuum oven at 180 °C. Toluene (anhydrous, 200 mL) was added to the dried silica nanoparticles. They were heated at 110 °C and stirred at 300 rpm for 10 min to form a well-dispersed suspension. After dissolving 2 g of organosilane in 50 g of toluene, the mixture was slowly dropped into the silica suspension at a rate of 1 mL/min. After the addition, the reaction mixture was maintained at 110 °C for 24 h with stirring.

The reaction mixture was centrifuged at 10,000 rpm for 20 min to remove toluene. Ethanol was added to wash the toluene remaining in the reaction product, and the mixture was dispersed again using a homogenizer speed of 1000 rpm for 2 min. This well-dispersed mixture was centrifuged at 10,000 rpm for 20 min to remove the solvent. Ethanol washing was repeated three times. Finally, the surface-modified silica nanoparticles were dried in a vacuum oven at 70 °C for 24 h. In this study, GPS-modified silica nanoparticles were named FS-EP, and APS-modified silica nanoparticles were named FS-NH.

### 2.3. Titration of Surface-Modified Silica Nanoparticles

To measure the concentration of epoxy and amine groups introduced onto the silica nanoparticles, the back titration method proposed by Hofen et al. was used [37]. First, HBr (0.1 N) and KHP (0.1 N) solution were prepared with acetic acid. HBr solution (0.1 N, 20 mL), MIBK (20 mL), acetic acid (20 mL) and 2–3 drops of methyl violet were added to an Erlenmeyer flask. The modified fumed silica (0.4 g) was dispersed in this solution using a magnetic bar at room temperature for 1 h. Titration was performed using a burette filled with 0.1 N KHP solution. The amount of 0.1 N KHP solution required to reach an endpoint was measured to calculate the surface concentration of epoxy or amine groups. All titrations were repeated at least three times.

### 2.4. Preparation of Silica-Modified Epoxy Adhesives

To make silica-modified epoxy adhesives with different volume fractions of silica nanoparticles, the density of silica nanoparticles was measured using a pycnometer (Ultrapyc 1200e, Quantachrome Instruments, Boynton Beach, FL, USA), and the results are displayed in Table 1.

Silica nanoparticles and YD-128 were premixed by a paste mixer (ARE-310, Thinky, Tokyo, Japan) at 2000 rpm for 10 min and then mixed with a three-roll mill (TR50, Trilos, San Ramon, CA, USA) for 3 min three times. The gaps between the rolls were 30 and 10 µm each. PTPU was added to this mixture and mixed with a paste mixer at 2000 rpm for 10 min. DICY was added 8 parts by weight per 100 parts of YD-128 (the ratio of amine to epoxy was 0.72), and accelerator was added 1 part [38,39]. The mixture was dispersed in a paste mixer (ARV-310, Thinky, Tokyo, Japan) at 2000 rpm for 3 min and then vacuum degassed under 1.0 kPa at 2000 rpm for 3 min. Table 2 shows the details of the composition of silica-modified epoxy adhesives.

### 2.5. Impact Wedge-Peel (IWP) and Lap Shear Tests

**Figure 1:**
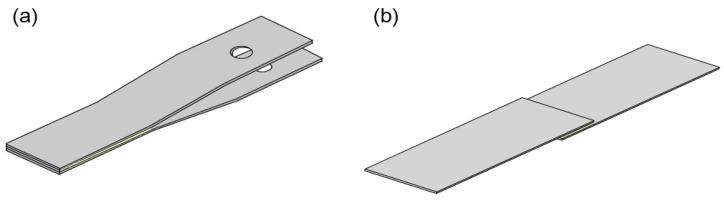
Impact wedge-peel (IWP) test specimen by Ford specimen design (**a**) and single lap joint (lap shear) test specimen (**b**).

The IWP test was performed according to the ISO 11343 standard to analyse the impact strength of the silica-modified epoxy adhesive. A strip for the IWP test was prepared based on the Ford specimen design (length: 90 mm, width: 20 mm, thickness: 1.6 mm, material: CR340), as shown in Figure 1a. A pair of strips were prepared and the surface of each strip was wiped off with acetone to remove dust. Adhesives were applied to a strip whose area was 30 × 20 mm^2^. The adhesive thickness was controlled by applying glass beads (200 µm thickness) on the adhesive layer. Two strips were stacked in the same direction. After fixing the overlapping strips with clamps, the strips were stabilized for 8 min and then cured at 180 °C for 20 min. The average adhesive layer size was 30 (±0.1) mm × 20 (±0.1) mm × 0.22 (±0.02) mm. The IWP tests were conducted using an impact drop tower (Model 7520, Instron, Norwood, MA, USA) at room temperature. The adhesive layer was cleaved by the wedge at a high test rate of 2.0 m/s. At least five samples were tested, and the average value was used.

The shear strength of the silica-modified epoxy adhesive was measured according to ISO 4587 standards [40]. The specimens of the lap shear test were prepared in dimensions (length: 25 mm, width: 25 mm, thickness: 1.6 mm, material: CR340), as shown in Figure 1b. In the same manner as the IWP test specimen, a pair of strips was prepared, and the surface of each strip was wiped off with acetone to remove dust. Adhesives were applied to the area of 25 × 12.5 mm^2^ on the strip, and a small amount of glass bead was sprinkled to maintain the adhesive thickness. After fixing the adhesive side of the overlapping strips with clamps, the strips were stabilized for 8 min and then cured at 180 °C for 20 min. The average adhesive layer size was 25 (±0.1) mm × 12.5 (±0.1) mm × 0.22 (±0.02) mm. Lap shear tests were conducted using a dual-column universal testing machine (Model 5969, Instron, Norwood, MA, USA) at room temperature. The crosshead speed was 5 mm/min. At least five samples were tested.

### 2.6. Single-Edged Notch Bending (SENB) Test

**Figure 2:**
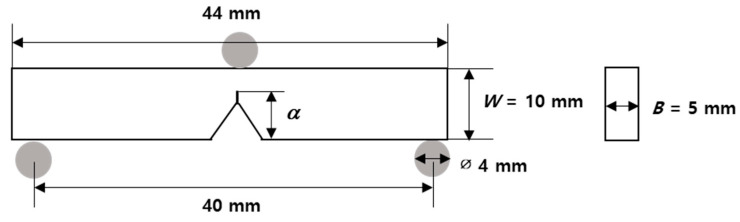
Dimension of the single-edged notch bending (SENB) specimen.

To measure the fracture toughness of silica-modified epoxy adhesives, the SENB test was conducted. SENB test specimens were prepared based on ASTM D5045-99, and their dimensions are displayed in Figure 2 [41]. All specimens were manufactured using silicone moulds. After pouring the adhesive on the silicone mould, it was degassed in a vacuum oven at 80 °C for 1 h and then cured in an oven at 180 °C for 20 min. After curing, it was separated from the silicone mould after cooling at room temperature. Finally, a precrack was made to obtain an accurate mode I fracture toughness (*K_IC_*) by removing residual stress around the crack tip. A three-point bending test machine (AG-X, Shimadzu, Kyoto, Japan) was used, and the crosshead speed was 10 mm/min. At least five samples were tested, and the average value was used.

The mode I fracture toughness (*K_IC_*) of silica-modified epoxy adhesives was calculated according to Equations (1) and (2) proposed by the ASTM D5045-99 standard.

(1) $$K_{IC}=\left(\frac{P_{Q}}{BW^{1/2}}\right)f\left(x\right),\;W=2B$$

(2) $$f\left(x\right)=6x^{1/2}\frac{\left[1.99-x\left(1-x\right)\left(2.15-3.93x+2.7x^{2}\right)\right]}{\left(1+2x\right){\left(1-x\right)}^{3/2}},x=\alpha /W$$

where *f* is a shape factor, *P_Q_* is a peak load, *B* and *W* are specimen thickness and width, respectively, and *α* is a crack length.

### 2.7. Dynamic Mechanical Analyser (DMA) Test

The glass transition temperature (*T_g_*) and viscoelastic properties of silica-modified epoxy adhesives were measured by DMA. The dimensions of the DMA specimen were 32 × 2.0 × 1.0 mm^3^ (length × width × thickness). The curing method was the same as that of the SENB specimens. The test was conducted using DMA 8000 (Perkin Elmer, Waltham, MA, USA) in tension mode and heated at 5 °C/min from −100 °C to 250 °C at a frequency of 1 Hz.

### 2.8. Other Characterizations

The surface functional groups of silica nanoparticles were characterized using X-ray photoelectron spectroscopy (XPS, K-Alpha, Thermo Fisher Scientific, Waltham, MA, USA). The morphological and particle size changes of silica nanoparticles before and after modification were analysed by transmission electron microscopy (TEM, K-Alpha, Thermo Fisher Scientific, Waltham, MA, USA). The fracture surface morphology of silica-modified epoxy adhesives was analysed by field-emission scanning electron microscopy (FE-SEM, JSM 6701F, JEOL, Tokyo, Japan). In addition, component analysis of different phases was conducted using energy dispersive spectrometry equipped with FE-SEM.

## 3. Results and Discussion

### 3.1. Comparison of Modified Silica Nanoparticles

**Figure 3:**
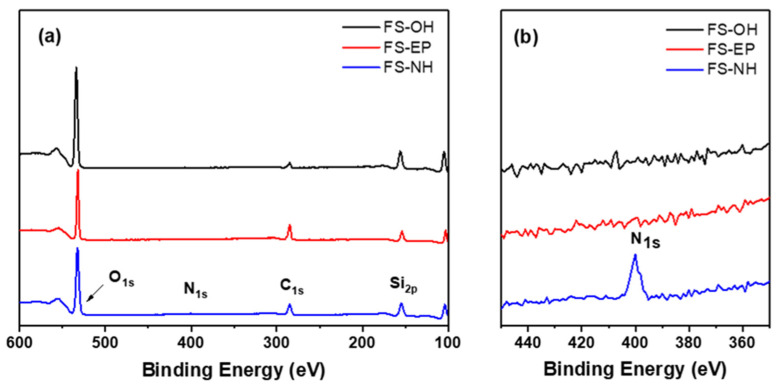
X-ray photoelectron spectroscopy (XPS) spectra (**a**) and N1s peak (**b**) of pristine, 3-Glycidyloxypropyl trimethoxysilane (GPS)- and 3-aminopropyl trimethoxysilane (APS)-modified silica nanoparticles.

The organosilane-treated silica nanoparticles were analysed by XPS to confirm their surface functional groups. As shown in Figure 3, FS-EP shows a stronger C1s peak at 270 eV than FS-OH, which is caused by carbon atoms in the propyl and glycidyloxy groups of GPS. Instead, FS-NH shows a new peak at 400 eV, which is assigned to N1s, due to the amine group of APS. These peaks confirm that the silica nanoparticles are well modified by GPS and APS treatments.

The quantitative analysis of epoxide and amine groups of modified silica nanoparticles was performed by the titration method using Equation (3), and the results are given in Table 3.

(3) $$\mathrm{Epoxy}\;\left(\mathrm{or}\;\mathrm{Amine}\right)\;\mathrm{Value}=\;\frac{\left(N\;\mathrm{of}\;\mathrm{KHP}\right)\times \left(V_{1}\right)-\left(N\;\mathrm{of}\;\mathrm{KHP}\right)\times \left(V_{2}\right)}{\mathrm{Weight}\;\mathrm{of}\;\mathrm{silica}}$$

where N is the normality of the KHP solution, V_1_ is the volume of the KHP solution consumed for titrating the standard solution, and V_2_ is the volume of the KHP solution consumed for titrating the modified silica-containing solution.

**Figure 4:**
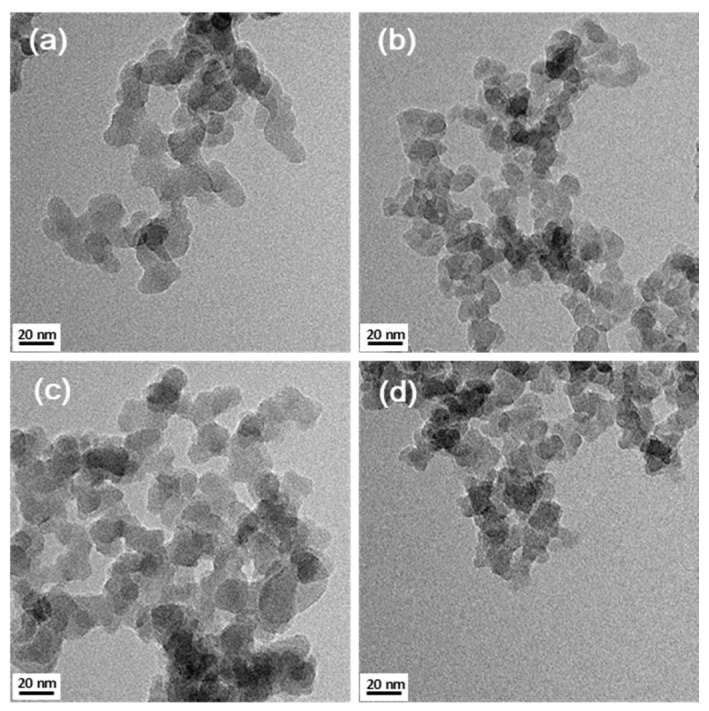
Transmission electron microscopy (TEM) images of (**a**) FS-polydimethylsiloxane (PDMS), (**b**) Hydrophilic fumed silica (FS-OH), (**c**) GPS-modified silica nanoparticles (FS-EP) and (**d**) APS-modified silica nanoparticles (FS-NH) (100,000× magnification).

Before and after the modification of silica nanoparticles, changes in shape and size were observed by TEM (Figure 4) and FE-SEM (Figure S1 in SI). Comparing Figure 4b–d, the shape and size did not change significantly before and after modification.

### 3.2. Thermomechanical Properties of Silica-Modified Epoxy Adhesives

**Figure 5:**
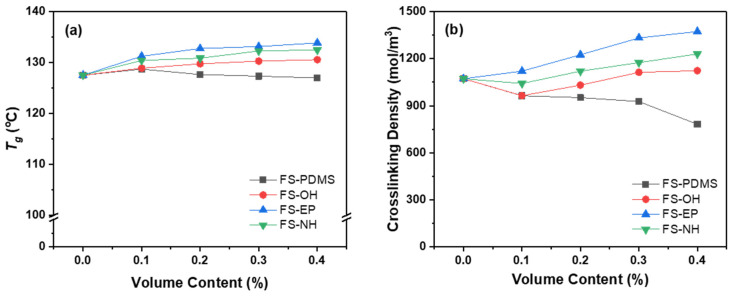
Dynamic mechanical analyser (DMA) measurement results by contents of silica nanoparticles: (**a**) glass transition temperature, and (**b**) crosslinking density.

The effect of silica nanoparticles with different surface functional groups on the *T_g_* and crosslinking density of epoxy structural adhesives was studied by DMA. Figure 5a shows that the *T_g_s* of epoxy adhesives seemingly increased with modified silica nanoparticles, and FS-EP-modified epoxy adhesives had the highest *T_g_*. However, FS-PDMS-modified epoxy adhesives showed slightly decreased *T_g_* with silica content. The crosslinking density of epoxy adhesives showed a similar trend with *T_g_s* (see Figure 5b). FS-EP induced the highest crosslinking density, followed by FS-NH. On the other hand, FS-PDMS constantly decreased the crosslinking density of epoxy adhesives with increasing silica content. These results imply that FS-OH, FS-EP and FS-NH can form covalent adducts during the curing reaction because their surface functional groups can react with an epoxy or an amine curing agent in the adhesive. These adducts increased the crosslinking density of epoxy adhesives and induced different phase separations and morphologies.

**Figure 6:**
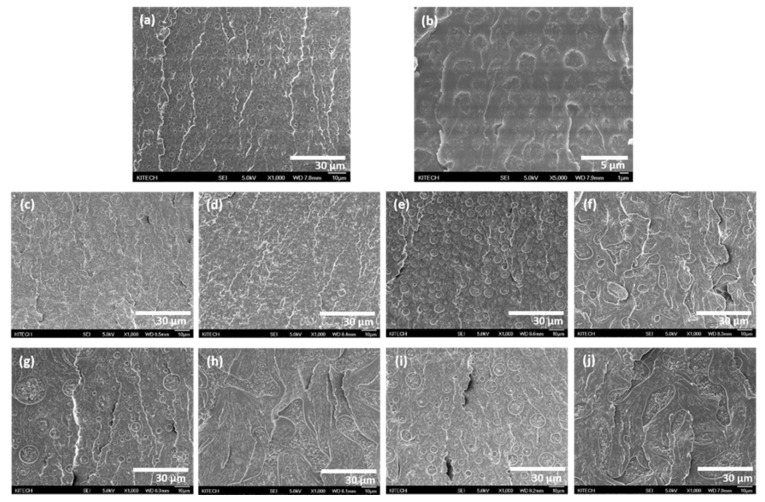
Scanning electron microscopy (SEM) images of the fracture surface of silica-modified epoxy adhesives by DMA specimens (1000× magnification except (**b**)): (**a**) control; (**b**) control (5000× magnification); (**c**) 0.1 vol% FS-PDMS; (**d**) 0.4 vol% FS-PDMS; (**e**) 0.1 vol% FS-OH; (**f**) 0.4 vol% FS-OH; (**g**) 0.1 vol% FS-EP; (**h**) 0.4 vol% FS-EP (**i**) 0.1 vol% FS-NH; (**j**) 0.4 vol% FS-NH.

Figure 6 shows SEM images of the fracture surface of epoxy adhesives modified with different silica nanoparticles. As shown in Figure 6b, the pristine epoxy adhesive showed approximately 2 µm-size PTPU domains in the epoxy continuous phase. As the volume content of silica nanoparticles increased, PTPU domains became larger, and the number of domains decreased. In particular, comparing the 0.1 and 0.4 vol% samples, FS-OH, FS-EP, and FS-NH showed different morphologies with silica nanoparticle contents. When 0.1 vol% silica nanoparticles were added, the domain size of PTPU was in the order of FS-EP > FS-NH > FS-OH > FS-PDMS. Epoxy adhesives with 0.4 vol% silica nanoparticles also showed the same order of domain size, but the domain shape was quite different from that of the 0.1 vol% samples. These morphological changes originate from the difference in the surface functional groups of silica nanoparticles. As previously mentioned, FS-OH, FS-EP and FS-NH can form covalent adducts through reaction with epoxy or a curing agent. These covalent adducts rapidly increase the network size and viscosity of the epoxy adhesives and then accelerate phase separation to give larger PTPU domains. It is well known that the faster the curing reaction is, the larger the domain and the fewer the number of domains [42].

Silica-epoxy adduct formation can be deduced from the distribution of silicon atoms in the epoxy and PTPU phases. The silicon atomic percentage by EDS (displayed in Table 4) shows that FS-PDMS and FS-OH existed in both epoxy and PTPU phases, whereas FS-EP and FS-NH did not exist in the PTPU phase. In particular, FS-PDMS preferred a PTPU phase to an epoxy phase, which was different from the other silica nanoparticles. This is because FS-PDMS had no surface functional groups to react with an epoxy or a curing agent, and the dispersion of FS-PDMS was simply determined by the interaction between epoxy or PTPU molecules. Accordingly, FS-PDMS decreased the crosslinking density because it simply disturbed the curing reaction.

Moreover, FS-OH preferred an epoxy phase to a PTPU phase due to its hydroxyl groups, which can react with epoxy groups. FS-EP and FS-NH also reacted with an epoxy resin or a curing agent and formed covalent adducts much more easily than FS-OH. They were preferentially included in the epoxy network structure via covalent bonding and effectively increased the crosslinking density compared to FS-OH and FS-PDMS.

### 3.3. Fracture Toughness of Silica-Modified Epoxy Adhesives

**Figure 7:**
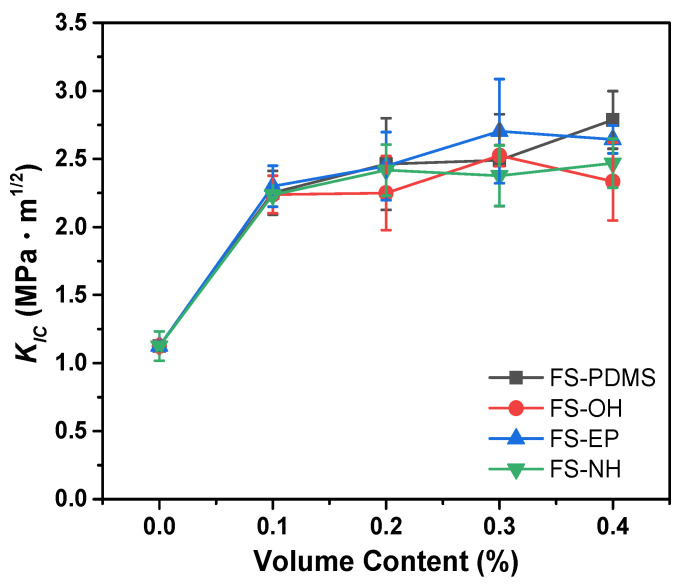
Mode I fracture toughness (*K_IC_*) of epoxy adhesives with different silica nanoparticles and contents.

Figure 7 shows the mode I fracture toughness (*K_IC_*) of silica-modified epoxy adhesives with different silica nanoparticles and volume contents. The fracture toughness of pristine epoxy adhesive increased remarkably with 0.1 vol% silica nanoparticles irrespective of surface functional groups. However, as the volume content of silica nanoparticles increased, the fracture toughness increased gradually, and it became difficult to distinguish the effect of surface functional groups of silica nanoparticles.

Considering that FS-PDMS decreased the crosslinking density of epoxy adhesives (see Figure 5b), FS-PDMS seems to increase the fracture toughness of an epoxy adhesive by increasing the network chain mobility. In general, as the crosslinking density decreases, the chain mobility increases, and the fracture toughness also increases.

Moreover, FS-OH, FS-EP, and FS-NH increased the crosslinking density of epoxy adhesives and increased fracture toughness. This may arise because cracks are effectively transferred and absorbed to the silica nanoparticles through the silica-epoxy adducts, although network chain mobility decreased due to silica-epoxy adduct formation.

### 3.4. Shear Strength of Silica-Modified Epoxy Adhesives

**Figure 8:**
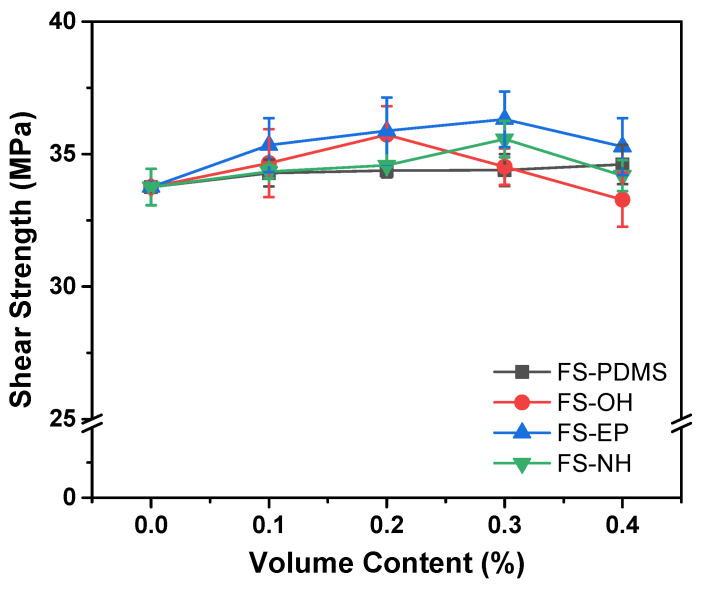
Lap shear test results of epoxy adhesives with different silica nanoparticles and contents.

During the lap shear test, the stress is concentrated at the ends of the overlaps and propagates along the adhesive layer. If the adhesive has strong interfacial adhesion with a substrate and high toughness, it will show a high level of shear strength. In Figure 8, FS-PDMS-modified epoxy adhesives showed almost constant shear strength, although the FS-PDMS content increased. This is different from the fracture toughness result, which shows that the fracture toughness of an epoxy adhesive increases with FS-PDMS content. Accordingly, the interfacial adhesion strength of an epoxy adhesive should decrease with FS-PDMS content, and this interfacial adhesion strength can be estimated by observing the fracture surface morphology.

**Figure 9:**
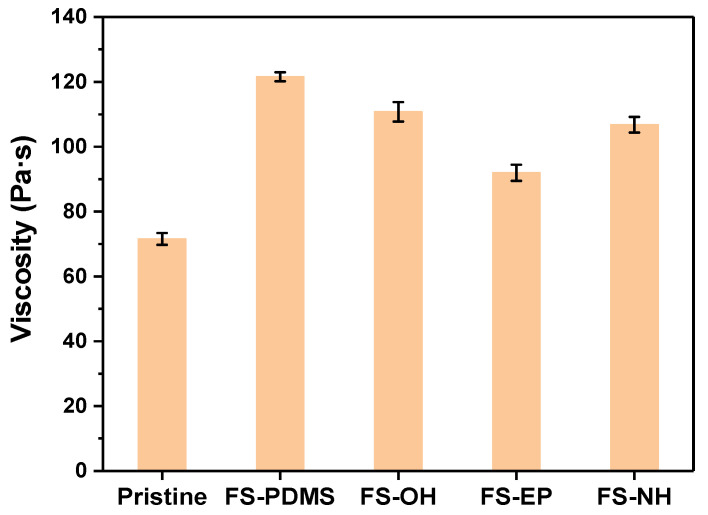
Viscosity of epoxy adhesives with different silica nanoparticles at 23 °C; silica content is 0.3 vol%.

The fracture surfaces of FS-PDMS-modified epoxy adhesives after the lap shear test are given in Figure S1 in the supplementary information. They showcase clean surfaces of test strips, which indicate interfacial failure. Since the interfacial adhesion is dominated by the wetting and specific interaction of an adhesive, the viscosity of silica-modified epoxy adhesives is measured. As shown in Figure 9, at 0.3 vol% silica nanoparticles, the viscosities of epoxy adhesives are 121.575 Pa·s with FS-PDMS and 91.981 Pa·s with FS-EP, which are higher than 71.585 Pa·s for pristine adhesive. Consequently, the low interfacial adhesion strength of PS-PDMS-modified epoxy adhesives is believed to be due to the increased viscosity.

Moreover, FS-EP increased the shear strength as well as the fracture toughness of epoxy adhesives. The fracture surface of the FS-EP-modified adhesive exposed strip surface was less than that of the FS-PDMS-modified adhesive (Figure S2 in SI). This implies that the interfacial adhesion of FS-EP-modified adhesive is stronger than that of FS-PDMS-modified adhesive. Comparing the fracture surfaces of FS-OH-, FS-EP-, and FS-NH-modified adhesives, the surface of a test strip is more exposed in the order of FS-OH, FS-NH > FS-EP, which is consistent with the order of viscosity, as shown in Figure 9.

Consequently, FS-PDMS increases the fracture toughness of an adhesive by lowering its crosslinking density but decreases its interfacial adhesion due to the increased viscosity. However, FS-EP increases the fracture toughness by transferring crack energy to silica nanoparticles and maintains interfacial adhesion because it induces a low increase in viscosity.

### 3.5. Impact Properties of Silica-Modified Epoxy Adhesives

**Figure 10:**
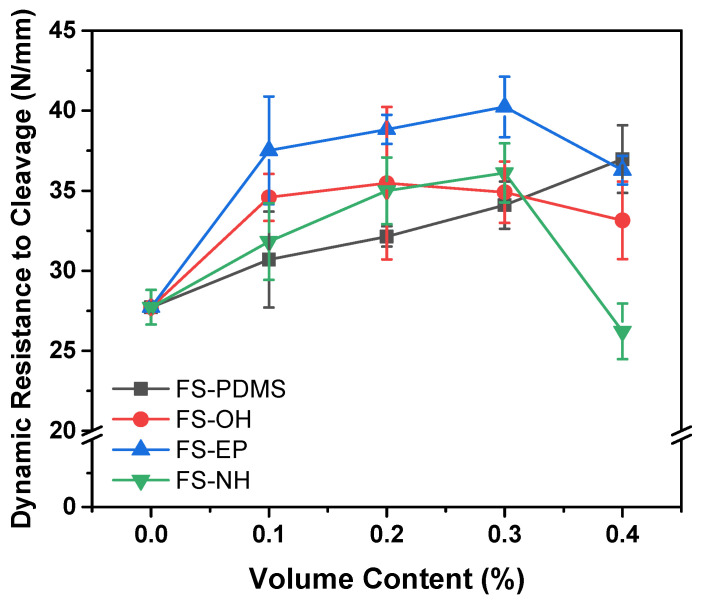
Dynamic resistance to cleavage of epoxy adhesives with different silica nanoparticles and content, obtained by the IWP test at room temperature.

Figure 10 shows the dynamic resistance to cleavage values of various silica-modified epoxy adhesives, which were obtained by IWP tests. Different from the fracture toughness and lap shear strength results, the dynamic resistance to cleavage values are apparently different according to the surface functional groups of silica nanoparticles.

FS-PDMS-modified epoxy adhesives showed that the dynamic resistance to cleavage values increased almost linearly with the content. Considering their fracture toughness and shear strength, it is mainly due to the increase in fracture toughness of the adhesives. However, FS-EP-modified epoxy adhesives showed quite different dynamic resistance to cleavage values, although they had similar fracture toughness as FS-PDMS-modified adhesives. As mentioned in the shear strength section, this difference is because FS-EP-modified epoxy adhesives have higher interfacial strength than FS-PDMS-modified adhesives. Moreover, the other silica nanoparticles, such as FS-OH and FS-NH, showed too small of a difference to interpret the effect of the surface functional groups.

When the silica content was 0.4 vol%, the dynamic resistance to cleavage values decreased as the shear strength decreased. Based on the fracture surface images (Figure S3 in SI), this decrease is due to the voids that increase with silica content. Considering that TGA curves showed FS-EP and FS-NH had obvious weight loss below curing temperature (180 °C) (Figure S4 in SI), these voids are thought to arise from the residues adsorbed on the silica surface during the silica modification. In particular, FS-NH-modified adhesive showed a drastically reduced value. This is partly because unreacted excess DICY decomposes to produce ammonia and other reactive amine compounds [43]. Therefore, more voids are observed as the content of FS-NH increases; as the content of FS-NH increases, more unreacted DICY remains.

## 4. Conclusions

In this study, silica nanoparticles with different surface functional groups were used to improve the high-energy impact resistance of epoxy structural adhesives, and the following conclusions were obtained.

Silica nanoparticles with nonreactive functional groups maintained the *T_g_* of epoxy adhesives but decreased their crosslinking density with increasing amounts. However, silica nanoparticles with reactive functional groups slightly increased the *T_g_* and crosslinking density of epoxy adhesives. In particular, silica nanoparticles with epoxy or amine functional groups obviously increased the crosslinking density, and they existed dominantly in epoxy phases rather than PTPU phases due to the formation of silica-curing agents or silica-epoxy adducts.

The fracture toughness of epoxy adhesives was increased by the addition of silica nanoparticles, but it was difficult to distinguish the effect of surface functional groups of silica nanoparticles. However, the dynamic resistance to cleavage values obtained by the IWP test definitely showed the effect of silica functional groups. This was because the IWP test was strongly affected by the interfacial adhesion between adhesives and IWP test strips.

The epoxy-functionalized silica nanoparticles showed higher shear strength and dynamic resistance to cleavage values than the amine-functionalized silica nanoparticles. Moreover, amine-functionalized silica nanoparticles significantly decreased the dynamic resistance to cleavage at 0.4 vol% due to the excess formation of voids. In this study, epoxy-functionalized silica nanoparticles were the most effective in improving the fracture toughness, shear strength and high-energy impact resistance of epoxy structural adhesives.

## Figures and Tables

**Table 1:** Density of silica nanoparticles.

Materials	Weight (g)	Volume (cc)	Density (g/cc)
FS-PDMS	0.2204	0.0758	2.9077
FS-OH	0.2939	0.0781	3.7634
FS-EP	0.5217	0.2242	2.3271
FS-NH	0.5762	0.2269	2.5389

**Table 2:** Composition of silica-modified epoxy adhesives.

Sample		YD-128 (g)	PTPU (g)	DICY (g)	Accelerator (g)	Silica (g)
Control		25	5.45	2	0.25	-
0.1 vol%	FS-PDMS	24.9750	5.4446	1.9980	0.2498	0.0820
	FS-OH					0.1061
	FS-EP					0.0656
	FS-NH					0.0716
0.2 vol%	FS-PDMS	24.9500	5.4391	1.9960	0.2495	0.1639
	FS-OH					0.2122
	FS-EP					0.1312
	FS-NH					0.1431
0.3 vol%	FS-PDMS	24.9250	5.4337	1.9940	0.2493	0.2459
	FS-OH					0.3183
	FS-EP					0.1968
	FS-NH					0.2147
0.4 vol%	FS-PDMS	24.9000	5.4282	1.9920	0.2490	0.3279
	FS-OH					0.4244
	FS-EP					0.2624
	FS-NH					0.2863

**Table 3:** Back titration results of silica.

Materials	Epoxide (Amine) Value (mmol/g)
FS-OH	0.025 ± 0.008
FS-EP	0.181 ± 0.031
FS-NH	0.222 ± 0.008

**Table 4:** EDS results of the fracture surface of silica-modified epoxy adhesives.

Silica	Vol%	Element	Epoxy Phase		PTPU Phase	
			Weight %	Atomic %	Weight %	Atomic %
FS-PDMS	0.1	C	72.99	78.29	73.69	78.89
		O	26.91	21.67	26.20	21.06
		Si	0.10	0.04	0.11	0.05
	0.4	C	70.21	75.93	74.15	79.49
		O	29.44	23.90	24.99	20.11
		Si	0.36	0.16	0.86	0.40
FS-OH	0.1	C	74.35	79.47	75.74	80.63
		O	25.47	20.44	24.19	19.33
		Si	0.18	0.08	0.08	0.03
	0.4	C	71.59	77.42	76.08	81.04
		O	27.04	21.95	23.44	18.75
		Si	1.37	0.63	0.47	0.22
FS-EP	0.1	C	73.83	79.04	76.26	81.06
		O	25.97	20.87	23.74	18.94
		Si	0.20	0.09	0.00	0.00
	0.4	C	72.52	78.01	75.25	80.19
		O	26.89	21.72	24.75	19.81
		Si	0.59	0.27	0.00	0.00
FS-NH	0.1	C	73.64	78.89	73.82	78.98
		O	26.09	20.99	26.18	21.02
		Si	0.27	0.12	0.00	0.00
	0.4	C	72.14	77.70	74.56	79.61
		O	27.20	22.00	25.44	20.39
		Si	0.66	0.30	0.00	0.00

## Data Availability

Data is contained within the article or supplementary material.

## Supplementary Materials

The following are available online at https://www.mdpi.com/2073-4360/13/3/469/s1. Fracture surfaces of epoxy structural adhesives, Figure S1: SEM images of (a) FS-PDMS, (b) FS-OH, (c) FS-EP and (d) FS-NH (×100,000 magnification), Figure S2: Fracture surfaces of epoxy structural adhesives obtained by lap tests: numbers in images mean volume content of silica nanoparticles, Figure S3: Fracture surfaces of epoxy structural adhesives obtained by IWP tests: numbers in images mean volume content of silica nanoparticles, Figure S4: TGA curves of surface-modified silica nanoparticles.
