# Supplementary material for: Comparative Study of the Impact Wedge-Peel Performance of Epoxy Structural Adhesives Modified with Functionalized Silica Nanoparticles
Source: Polymers (Basel). 2021 Feb 2;13(3):469. doi: 10.3390/polym13030469 (PMC7867298; doi:10.3390/polym13030469)
Supplement: Supplementary file 1 [file polymers-13-00469-s001.pdf]

polymers

Supplement Information

Comparative Study of the Impact Wedge-Peel Performance of Epoxy Structural Adhesives by Surface Functionalized Silica Nanoparticles

Gyeong-seok Chae [1,2], Hee-woong Park [1], Kiok Kwon [1] and Seunghan Shin [1,2,*]

Before and after the modification of silica nanoparticles, changes in shape and size were observed by FE-SEM in Figure S1.

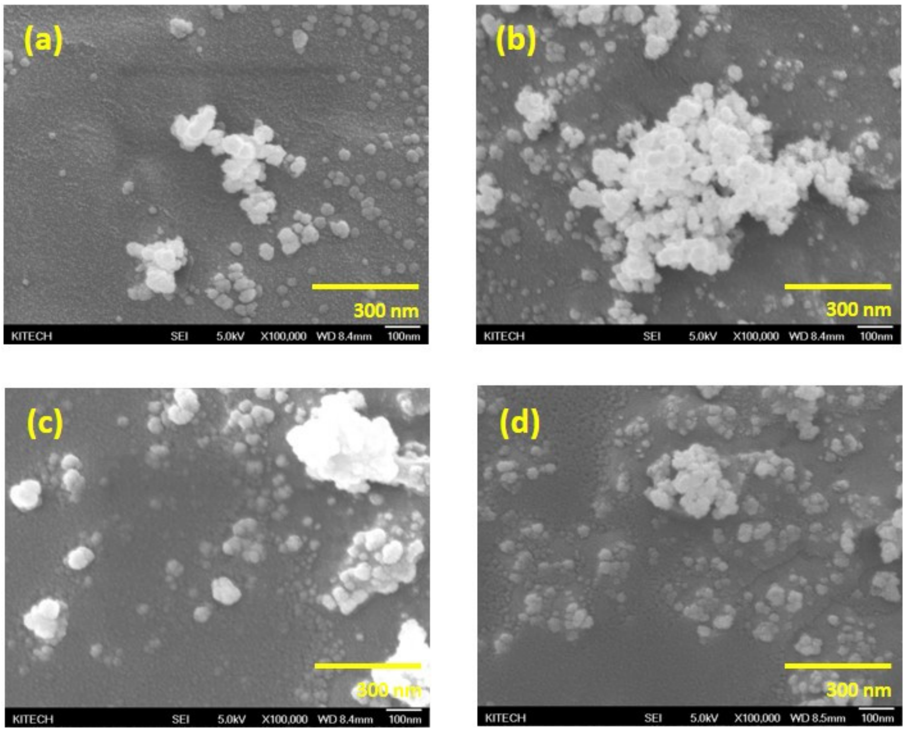

Figure S1. SEM images of (**a**) FS-PDMS, (**b**) FS-OH, (**c**) FS-EP and (**d**) FS-NH (×100,000 magnification).

The fracture surfaces of test specimens after lap shear and IWP test are displayed in Figures S2 and S3, respectively. The fracture surfaces were listed in the order of FS-PDMS, FS-OH, FS-EP, and FS-NH according to the content of silica nanoparticles.

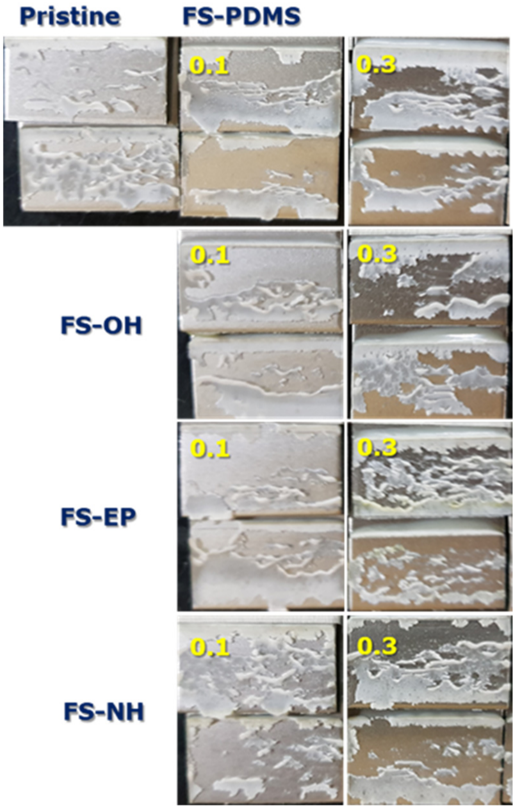

Figure S2. Fracture surfaces of epoxy structural adhesives obtained by lap shear tests: numbers in images mean volume content of silica nanoparticles.

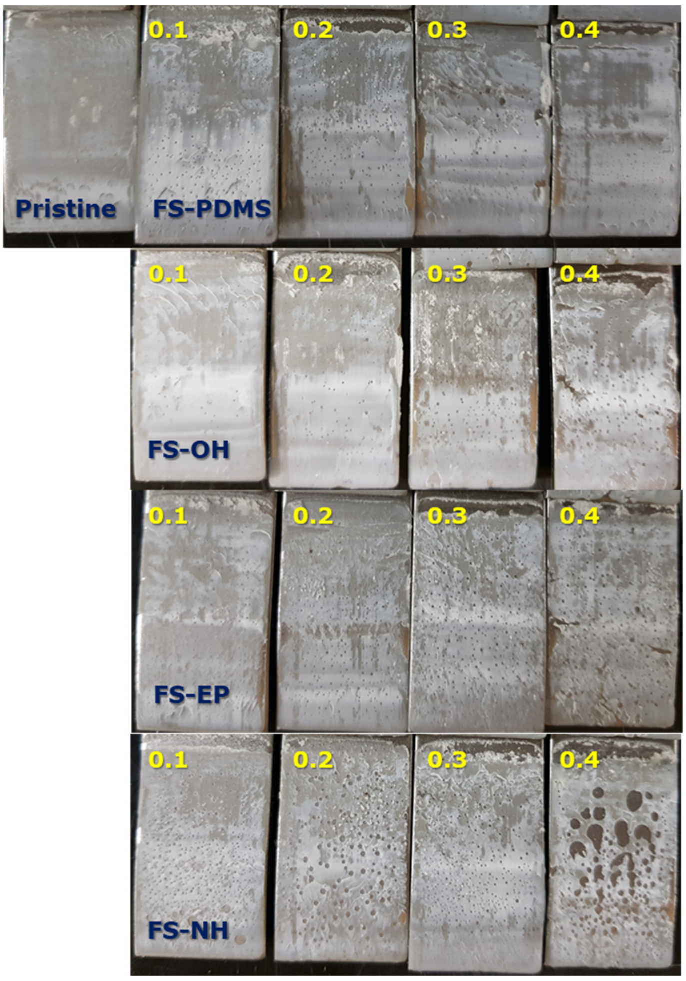

Figure S3. Fracture surfaces of epoxy structural adhesives obtained by IWP tests: numbers in images mean volume content of silica nanoparticles.

The moisture content and organic content of silica nanoparticles with different surface groups were measured by TGA. All samples were measured while heating from 30 to 800 °C by 20 °C/min.

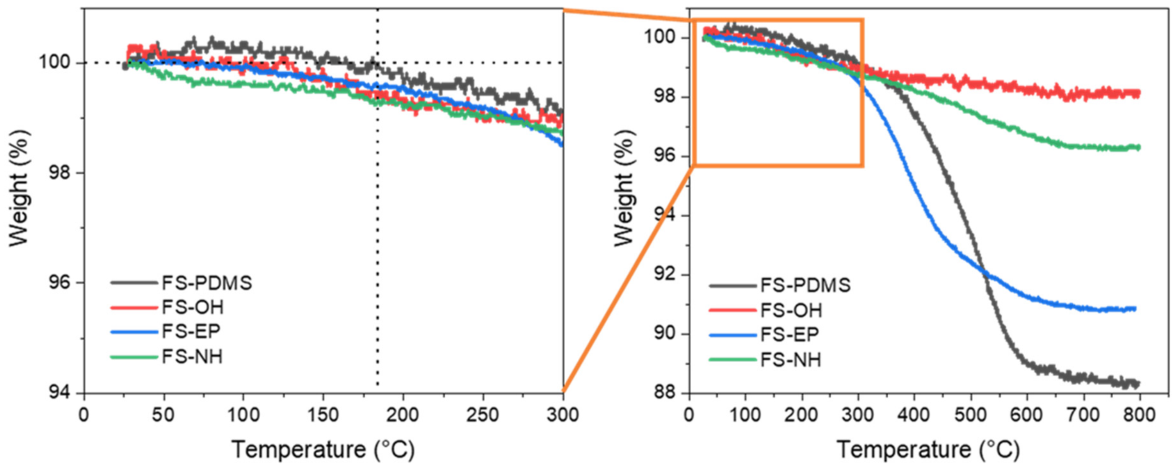

Figure S4. TGA curves of surface-modified silica nanoparticles.
